# The Effect of Transposable Element Insertions on Gene Expression Evolution in Rodents

**DOI:** 10.1371/journal.pone.0004321

**Published:** 2009-02-02

**Authors:** Vini Pereira, David Enard, Adam Eyre-Walker

**Affiliations:** Centre for the Study of Evolution, School of Life Sciences, University of Sussex, Brighton, United Kingdom; Georgia Institute of Technology, United States of America

## Abstract

**Background:**

Many genomes contain a substantial number of transposable elements (TEs), a few of which are known to be involved in regulating gene expression. However, recent observations suggest that TEs may have played a very important role in the evolution of gene expression because many conserved non-genic sequences, some of which are know to be involved in gene regulation, resemble TEs.

**Results:**

Here we investigate whether new TE insertions affect gene expression profiles by testing whether gene expression divergence between mouse and rat is correlated to the numbers of new transposable elements inserted near genes. We show that expression divergence is significantly correlated to the number of new LTR and SINE elements, but not to the numbers of LINEs. We also show that expression divergence is not significantly correlated to the numbers of ancestral TEs in most cases, which suggests that the correlations between expression divergence and the numbers of new TEs are causal in nature. We quantify the effect and estimate that TE insertion has accounted for ∼20% (95% confidence interval: 12% to 26%) of all expression profile divergence in rodents.

**Conclusions:**

We conclude that TE insertions may have had a major impact on the evolution of gene expression levels in rodents.

## Introduction

Although transposable elements (TEs) are selfish genetic elements, primarily interested in their own reproduction, they have contributed substantially to genome evolution. Besides contributing large amounts of DNA to many genomes, including at least 40% of the human genome [1], they have also provided new genes [2], exons [3], [4] and motifs involved in chromosome structure [5]. There are also several examples in which a TE now forms part of the machinery regulating gene expression [5], [6], [7], [8]. However, recent genomic analyses suggest that TEs may play a much more general role in the evolution of gene regulation as McClintock [9] and Britten and Davidson [10] first suggested. Analyses of non-genic sequences, which are conserved across distantly related species, show that a considerable fraction are of TE origin [11], [12], [13], [14], [15]. Since some conserved non-genic sequences (CNG) appear to be involved in gene regulation [16], this suggests that TEs may have contributed substantially to the evolution of gene expression. In fact one CNG sequence, which resembles a TE, has been recently shown to regulate the expression of the human gene *IsL1* [17]. Additionally, TEs appear to be associated with many DNA hyper-sensitivity sites in the human genome [18].

However, while beguiling, these observations do not demonstrate that TE insertions themselves contribute directly to the evolution of gene expression. Many genomes are littered with TEs, so if a gene regulatory element is to evolve, it is quite likely to do so within a TE sequence [2], [18]. So it may not be the insertion itself which alters expression, but mutations after the insertion within the element. In fact several of the classic examples, in which it has been shown that an element is involved in regulation, appear to have evolved the critical transcription factor binding sites after insertion. For example, the endothelin-B receptor gene has an long tandem repeat element in the promoter which contains two putative placenta specific transcription factor binding sites, but neither of these are present in 60 related HERV-E TEs [19].

Here we investigate whether new TE insertions affect gene expression patterns by testing whether there is a correlation between the divergence of gene expression profiles between species and the number of new TE elements that have been integrated near genes. If TE insertions cause changes in expression then we would expect a correlation between expression divergence (ED) and the numbers of new TEs. If, however, gene control elements simply evolve within TE elements then we would expect ED to be correlated to the numbers of new and ancestral TEs, but only if certain types of TEs tend to evolve into regulatory sequences.

Two groups have previously examined the correlation between ED and TE insertion. First, Marino-Ramirez et al. [18] showed that genes with a DNase I hypersensitive site had higher ED when the site was derived from a TE element than when it was not; since they were comparing human and mouse it was very likely that many of the TEs they were considering in humans were insertions that had occurred since human and mouse diverged. Second, Urrutia et al. [20] have reported that ED between human and mouse is correlated to the numbers of new Alu elements in humans, although the direction of the correlation depended upon the measure of expression divergence employed. However, there are several differences between their analysis and the one presented here. First, they only consider the numbers of new TE insertions along the primate lineage, not the rodent lineage. Second, they only consider Alus. Here we test whether ED between mouse and rat is correlated to the numbers of new TE insertions of three types. We show that there is a positive correlation between ED and the numbers of new, but not ancestral, TE insertions and we estimate that the contribution of TEs to gene expression is substantial.

## Results

To investigate whether new TE insertions affect gene expression patterns we assembled a dataset of 3072 orthologous genes from mouse and rat for which we had gene expression data across 17 tissues in both species and aligned genomic sequences. For each gene we scored the number of new LTR, SINE or LINE insertions in one of 8 regions in and around the gene sequence: 0–2 kb, 2–10 kb and 10–20 kb upstream and downstream of the start and end of transcription, the first 2000 bp of the first intron and the last 2000 bp of the last intron. We scored a sequence as having a new TE if the mouse sequence contained a TE that was absent in rat and the genomic alignment had a gap in the rat sequence that matched the length of TE sequence in mouse, and *vice versa*. Although, it seems unlikely, these could also be cases of perfect deletion. Unfortunately without a close outgroup it is impossible to confirm whether we are dealing with insertions or perfect deletions, except for LTR elements, where we can determine the age of the elements by considering the divergence between the long terminal repeats – they are identical on insertion of the element. We found that 95% of LTRs had a divergence between their repeats that was lower than the average divergence between mouse and rat, which suggests that the vast majority of LTRs, which we scored as new insertions, have indeed inserted since mouse and rat diverged.

### Numbers of new elements

The total number of new TE insertions for each TE type and region are given in table 1. From this it is apparent that new TE insertions are quite common in mouse and rat; the majority of genes have a new LTR, SINE and LINE insertion within 20 kb of the coding region. SINEs appear to be the most active TE family in rodents followed by LTR elements and then LINEs. This is in contrast to the initial analysis of the mouse genome sequence in which LINEs appeared to be the most active element, with LTRs and SINEs showing similar levels of activity [21]. The discrepancy may in part be due to the size of the element; i.e. the activity of an element was quantified as the proportion of the genome contributed by TE activity in the mouse genome analysis.

**Table 1:** The numbers of new TE insertions in each genomic region in mouse and rat.

	LTR	SINE	LINE
5′UTS (10–20 kb)	947	5848	594
5′UTS (2–10 kb)	814	5119	462
5′UTS (0–2 kb)	109	1065	80
First intron (0–2 kb)	27	564	40
Last intron (0–2 kb)	56	604	70
3′UTS (0–2 kb)	149	1012	109
3′UTS (2–10 kb)	712	4438	457
3′UTS (10–20 kb)	813	5660	557

### Regression analysis

To investigate whether gene expression divergence correlates to the number new TEs we regressed the Box-Cox transformed expression divergence (ED) against the numbers of new TE insertions of three types in 8 regions. Expression divergence was measured as the Euclidean distance between the log of the relative abundances across the 17 tissues, as measured in two microarray experiments. As such ED measures changes in the pattern of expression across tissues rather than changes in absolute expression level; the latter is difficult to measure across species because the gene may bind to the probes on the microarray for one species more tightly than it does to the probe in another species [22]. We also measured expression divergence as the angle between the expression levels, the angle between the log expression values and the cross entropy; these measures gave qualitatively similar results, which is perhaps not surprising since these measures are highly correlated after a Box-Cox transformation (correlation between the Euclidean distance and the Angle = 0.86, the Angle of the log expression values = 0.93, and the cross entropy = 0.98). We did not use the correlation coefficient as a measure of expression divergence since there are problems with this measure for genes that are uniformly expressed (unpublished results).

Although many of the gradients are positive in the multiple regression we find no evidence that ED is significantly correlated to the numbers of new TEs (p = 0.49). However, our expression dataset is dominated by tissues which come from various parts of the brain and these show an idiosyncratic pattern of evolution. Liao and Zhang [22] found that when they clustered tissues according to the similarity of their expression profiles, mouse brain tissues clustered together to the exclusion of the human brain tissues, which also cluster together. In contrast, for other tissues they observed mouse and human clustering together; for example, mouse heart was most similar to human heart. This is as we might expect; we expect a tissue to be defined by its pattern of gene expression (although not necessarily at the developmental stage that was sampled). We observe a similar pattern for mouse and rat tissues (Figure S1); furthermore we note that brain tissues seem to have evolved relatively slowly in mouse and rat, as others have previously noted [23].

**Figure 1:**
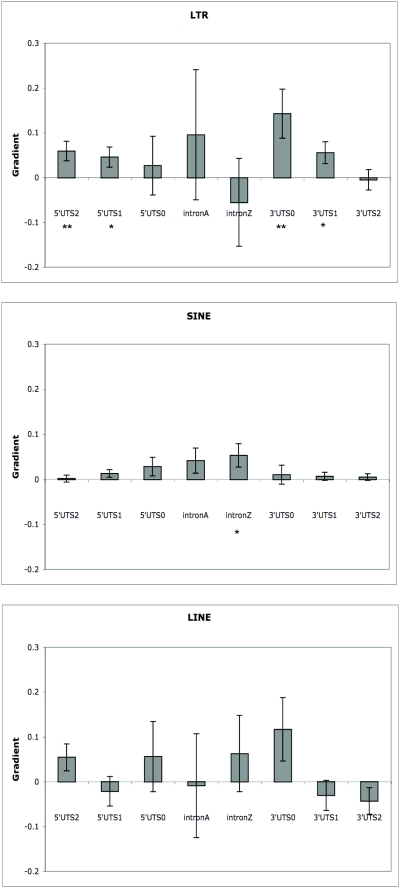
The gradients, with standard errors, from a multiple regression of ED across the 9 tissues against all TE and region combinations. Regions are abbreviated as follows: UTS0 = UTS(0–2 kb), UTS1 = UTS(2–10 kb), UTS2 = UTS(10–20 kb), intronA = first intron, intronZ = last intron. Significance is indicated as follows: * p<0.05 ** p<0.01.

The fact that almost 50% of the tissues in our sample evolve slowly and in a concerted fashion might disguise factors that affect the divergence of gene expression more generally. We therefore repeated our analysis excluding the 8 brain tissues. The results from this subset of data are very different to those observed using data from all tissues. First, the multiple regression is highly significant (p<0.0001), with several of the gradient terms being individually significant (Figure 1). In all but one case the correlation between ED and the numbers of new LTR and SINEs is positive and several of the LTR terms and one of the SINE terms is individually significant. In contrast LINEs show no consistent pattern and none are significant.

To further investigate the main factors we ran stepwise regression models. Both forward and backward models gave the same final model that includes 5 terms with nominal significance of p< = 0.05 (Table 2). The model is dominated by the effects of LTR and SINE elements with the strongest term being for LTR elements integrated into the 3′UTS (0–2 kb).

**Table 2:** The regression coefficients from a stepwise multiple regression of ED against TE-region.

TE	Region	Coefficient	p-value
LTR	3′UTS(0–2 kb)	0.150	0.0061
LTR	5′UTS(10–20 kb)	0.059	0.0067
SINE	Last intron	0.055	0.033
SINE	First intron	0.054	0.050
LTR	3′UTS(2–10 kb)	0.052	0.030
LTR	5′UTS(2–10 kb)	0.045	0.0067
SINE	5′UTS (2–10 kb)	0.020	0.0082

New terms were included (or excluded) from the model such that all factors in the model were nominally significant at p< = 0.05.

### Causality

Although, ED is significantly correlated to the number of new TE insertions, this correlation could be non-causal – it might be that new TEs tend to integrate into genes that undergo fast expression evolution, or that they are preferentially retained in such genes. However, if this was the case then we should observe a correlation between ED and the numbers of TEs that integrated prior to the divergence of mouse and rat, unless the pattern of integration has changed recently. To investigate the matter further, we tabulated the numbers of each TE type that were shared between mouse and rat in each region, and re-ran the regression analysis.

Including the number of shared TEs in our model makes little difference. Overall the model remains highly significant (p<0.0001) but only one shared TE term is significant, this is for shared SINEs in the 3′UTS (0–2 kb) region. Stepwise regression returns almost exactly the same model as before, with only one significant shared-TE term, SINEs in 3′UTS (0–2 kb) (Table 3). If we regress ED against the numbers of shared TEs by themselves the model is nearly significant (p = 0.0512), but this seems to be due solely to the SINEs in 3′UTS (0–2 kb) term, since removing this eliminates any hint of significance. The lack of a correlation between ED and the numbers of ancestral TEs is not due to sample size because there are many more shared TEs than lineage specific TEs.

**Table 3:** The regression coefficients from a stepwise multiple regression of ED against the numbers of new and shared TEs in each region.

TE	Region	Coefficient	p-value
LTR-new	3′UTS(0–2 kb)	0.156	0.0043
LTR-new	5′UTS(10–20 kb)	0.061	0.0052
SINE-new	Last intron	0.054	0.037
LTR-new	3′UTS(2–10 kb)	0.053	0.029
LTR-new	5′UTS(2–10 kb)	0.045	0.046
SINE-shared	3′UTS(0–2 kb)	0.033	0.0058
SINE-new	5′UTS (2–10 kb)	0.018	0.015

New terms were included (or excluded) from the model such that all factors in the model were nominally significant at p< = 0.05.

Two more arguments also support a causal link between new TE insertions and ED. First, we note that all the significant correlations are positive, which is as we would expect if the correlations were causal. In contrast if the correlations were non-causal in origin then we might expect some of the correlations to be negative. Second, if TE insertions directly affect gene expression patterns then we might expect there to be a negative correlation between the regression coefficient and the density of TE elements in each genomic region. This is because if TEs tend to affect gene expression when they insert in a particular region then they will generally be deleterious and therefore removed by natural selection. This correlation is observed for SINE elements (Spearman's rank correlation r = −0.81, p = 0.015) and for LTR elements if we restrict the analysis to the regions in which the number of new LTR elements correlates significantly to ED, although the latter is not significant (r = −0.8, p = 0.20).

### Quantification

Generally within genomic analyses we consider the biological significance of a correlation by considering the proportion of the variance explained; if the proportion variance explained is very small, as it is here (2%), then we might regard the correlation as being biologically irrelevant. However, in the present context we are less interested in the variance explained, than in the average effect that TEs have on gene expression evolution; these can be very different. For example, consider the effects of smoking on the risk of developing lung cancer. We are primarily interested in the increase in the risk that smoking represents, as measured by the gradient or odds-ratio, not the proportion of the variance explained. It is perfectly possible for TEs to be the sole cause of ED, and yet explain little of the variance in ED. For example, it might be that housekeeping genes evolve slowly because only rarely is a change in expression advantageous, whereas tissue specific genes evolve rapidly; if TEs are the only mutation that affects gene expression, there will be a correlation between ED and TE numbers, but the variance explained will be very small, because the variance in ED is dominated by genes having different rates of ED. We therefore estimated the proportion of ED that is due to TEs in the following manner. The intercept of a regression model represents the estimated ED when there have been no TE insertions; therefore one minus the intercept over the mean represents the average proportion of ED that is due to TE insertion.
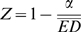
(1)where α is the intercept from the regression model. This is equivalent, in spirit, to multiplying the gradient for each TE-region combination by the average number of TEs that a gene has in each region and then dividing this by the mean of ED. Unfortunately, the value of *Z* is dependent on the way in which ED is measured. For example, if we regress the Box-Cox transformed value of ED against the 24 TE-region combinations and then back-transform the mean and intercept to the Euclidean distance, the estimate of Z is 9.9%; but if we back-transform the mean and intercept to the square-root of the Euclidean distance Z = 5.5%. We therefore need to know the natural scale over which ED evolves. We believe that this scale is the one over which ED increases linearly with time under a realistic evolutionary model. Khaitovich et al. [24] have previously shown that the square of the difference in log expression level, for a single gene in a single tissue, between two species increases linearly with time under a simple random walk model, which implies that the square of the Euclidean distance will also be linear with time. We have demonstrated by simulation that this simple relationship also holds even when we calculate relative abundance values (Text S1 and Figure S2). If we transform the mean and intercept from the regression model using the Box-Cox transformed data to the square of the Euclidean distance we estimate that on average TE insertions account for 19% of all expression divergence (95% confidence intervals from bootstrapping: 10%, 26%). This is likely to be an underestimate for two reasons. First, we have shown that *Z* tends to be underestimated when the regression is performed on the Box-Cox transformed data (Text S1 and Figure S3). Second, we have ignored error in the measurement of expression levels.

## Discussion

We have shown that expression divergence between mouse and rat is significantly correlated to the numbers of new TEs integrated near genes, but that it is essentially uncorrelated to the numbers of TEs that are shared between mouse and rat. These results suggest that new TE insertions are significantly involved in the evolution of gene expression in these species. Furthermore, we estimate that ∼20% of all expression divergence is a consequence of TE insertion.

Our results are largely consistent with those of Urrutia et al. [20] who found significant correlations between various measures of ED between human and mouse and the numbers of Alu elements; note that Alus are primate specific so this is equivalent to finding a correlation between ED for human-mouse and the numbers of new Alu elements in the primate lineage. However, while all our measures of ED gave similar results, Urrutia et al. [20] found that most measures of ED gave a positive correlation, but that the Euclidean distance gave a significantly negative relationship. The reason for this discrepancy is not obvious; it may be due to statistical differences, they did not transform their Euclidean distance values to be normal, or there might be a genuine difference between the way in which expression evolves in primates and rodents, and the role that TEs play in that divergence. We believe our analysis has some advantages over that of Urrutia et al. [20]; we study expression divergence over a relatively short timescale and correlate this to the numbers of new TEs, of three different types, in both lineages; Urrutia et al. [20] look at ED over a much larger time-scale and they only consider new TE insertions of one type along one lineage. We therefore believe that the pattern we observe is likely to reflect the true pattern; that ED is positively correlated to the numbers of new LTR and SINE insertions.

LTR and SINE elements appear to have the strongest effects on expression divergence in rodents in our analysis. This is broadly consistent with the pattern seen in TEs that are known to be part of promoter elements. Of the TEs which have been shown to be involved in gene regulation in mammals, the majority are SINEs, with a moderate number of LTRs and a few LINEs and DNA transposons [7], [8], [18]. In contrast the proportion of CNGs that are derived from TEs are dominated by LINEs [13] or DNA elements [12] depending on the definition of a CNG, although SINEs are also sometimes implicated [11]. Silva et al. [11] found that *mir* SINE and L2 LINE elements were significantly more conserved that expected between humans and mouse, whereas Lowe et al. [13] found that more than 50% of all TE derived CNG nucleotides come from LINEs, with ∼25% coming from SINEs and a small proportion from LTR and DNA elements. In contrast, Kamal et al. [12] found that the majority of bases in the 115 examples of CNGs that overlap TEs come from one DNA element, *mer121*. The differences between our results and those derived from the analysis of CNGs are likely to be due to three main factors. First, it is known that TE activity varies through time with some families being active while others are relatively quiescent. Since Lowe et al. [13] and Kamal et al. [12] surveyed CNGs that are shared across distantly related animals, their sample is likely to be dominated by older TE families, whereas we are considering families which have been active in the recent past. Second, it is evident that some TE families are more likely to be co-opted into functional roles; this seems to be the case for *mer121* which shows a similar average level of divergence to *mer119*, but has a higher proportion of cases in which part of the sequence has been very highly conserved [12]. Finally, it is possible that LINEs, which often constitute a large part of a genome, tend to evolve to become a regulatory element after insertion, rather than generating regulatory novelty on insertion.

The lack of any LINE effects is perhaps surprising given the experimental work of Han and Boeke [25]. They showed that integrating a complete or partial L1 element into an intron can significantly reduce expression. The lack of a LINE effect in our analysis might be due to the limited amount of intron sequence we analysed, although this was large enough to detect SINE effects, or it might be that this form of regulation is rarely used; there are very few examples of this type of regulation in nature [26]

The recent observation that many conserved non-genic sequences bear a resemblance to known transposable elements [12], [13], [14], [15] has supported the suggestion that TEs may have an important role in the evolution of gene regulation. However, this observation is open to two interpretations; either the TE insertion itself alters the expression profile of the gene, or mutations in the element after it has inserted change the profile. Our results strongly suggest that it is the former because our insertions are very recent and we observe no correlation with ancestral TEs. It therefore seems that new insertions significantly alter gene expression patterns and that TEs therefore play a direct and significant role in the evolution of gene expression.

## Materials and Methods

### Gene expression data

We obtained microarray gene expression data for mouse and rat from the experiments of Su et al. [27] and Walker et al. [28] respectively. The platform annotation was used to obtain the Entrez Gene ID of each gene targeted by the probesets and these were used to identify orthologous genes using R packages, RNOhomology and MMUhomology (version 1.12.0), which contain data from the NCBI Homologene project (http://www.ncbi.nlm.nih.gov/HomoloGene).

The mouse and rat expression experiments surveyed expression levels across 30 and 61 tissues respectively of which 17 were in common. These were amygdala, bone marrow, cerebellum, cerebral cortex, dorsal root ganglion, dorsal striatum, frontal cortex, heart, hippocampus, hypothalamus, kidney, large intestine, pituitary, skeletal muscle, small intestine, spleen and thymus. Each tissue experiment had two replicates in mouse and a varying number of replicates in rat; some genes were also matched by multiple probesets. To obtain an average across experiments and probesets we processed the data as follows. We obtained raw CEL files of gene expression levels from the NCBI Gene Expression Omnibus database (http://www.ncbi.nlm.nih.gov/projects/geo/). We normalized the results from the mouse and rat arrays separately using the RMA algorithm [29] as implemented in Bioconductor [30]. We then averaged the expression of each gene in each tissue across experiments and probesets.

### Expression divergence

Although, the intensity with which a particular probe binds a particular mRNA is proportional to the expression of the gene, the probe for a particular gene may bind the mRNA more tightly in one species than other, and hence give the impression that absolute levels of expression are different between species. Hence, we followed the suggestion of Liao and Zhang [22] and calculated relative abundance (RA) values; this is the expression of a gene in a species and tissue divided by the summed expression of that gene across all sampled tissues in that species. These values represent the relative abundances of the gene across tissues. To measure the difference between the expression profiles of a gene in mouse and rat we calculated the Euclidean distance between the log of the RA values. We also calculated the angle between the expression values and the log expression values as an alternative measure of expression divergence and obtained very similar results (results not presented). We also used the cross-entropy. We did not use Pearson's correlation coefficient, because as will show elsewhere, there are problems with this measure for genes that are uniformly expressed.

### Identifying new insertions

To identify TEs which are likely to be new insertions in either mouse or rat, since these species diverged, we obtained RepeatMasker [31] annotation of TE sequences and genomic alignments from the UCSC Genome Browser [32] database for the mouse NCBI 36 (UCSC mm8) and the rat RGSC 3.4 (UCSC rn4) genome assemblies. A single TE insertion can appear as several pieces within the RepeatMasker annotation for a number of reasons. First, RepeatMasker annotates some TE types, such as LTR elements, as several separate entities. Second, evolution within the TE can break up the homology between the consensus and the TE sequence. And third, TEs sometimes insert into other TEs breaking them into two parts. We therefore applied a new algorithm, implemented in the program ReAnnotate [33], to join these disparate parts into single TE elements where possible, a process we call *defragmentation*. In short the algorithm connects TE fragments to each other that are in the same orientation and co-linear. Having defragmented the TEs we inferred a new TE insertion when a TE annotated in one species fell (almost) exactly within a gap in the other species; we allowed for some leeway in the accuracy of the annotation of the TE, by allowing it to be either 20 bp shorter or longer than the gap at either end. We also annotated as new insertions TEs which fell within another TE which was itself a new insertion. We also tabulated the numbers of TEs that were shared by mouse and rat in orthologous positions.

We considered the effects on gene expression of four categories of TEs: LTR (Long Terminal Repeat retrotransposons and endogenous retroviruses), SINE (Short Interspersed Nuclear Elements), LINE (Long Interspersed Nuclear Elements), and DNA transposons. However, DNA transposons are relatively quiescent in rodents [21] so there are very few new insertions. They were therefore excluded from further analysis.

Insertions were allocated to one of eight regions: the first 2000 bp of the first intron, the last 2000 bp of the last intron, and the 5′ and 3′ untranscribed (UTS) regions broken into the following regions upstream and downstream of the gene: 0–2 kb, 2–10 kb and 10–20 kb. By untranscribed sequence we refer to the region upstream of the transcription start site and downstream of the transcription termination site. We considered the first 2000 bp upstream and downstream of genes along with the first 2000 bp of the first intron because these regions have been shown to be subject to selective constraint in rodents [34] and we therefore expected to be able to detect the effects of TE insertion most readily. If a new insertion overlapped two regions it was assigned to the region nearest the protein coding sequence. For example, if a mouse specific SINE overlapped the region 1900 to 2200 bp upstream of the start of transcription it would be allocated to the 5′ UTS (0–2 kb) category. We also considered new TE insertions in the untranslated regions, but we found very few of these and they were therefore subsequently dropped from the analysis.

### Transcript structures

Transcript structures were obtained by using the Entrez Gene IDs to query Ensembl v.39 (http://ensembl.org) annotation [35]. Complete information was unavailable for 903 genes which were excluded from further analysis to yield a final dataset of 3072 genes. If a gene had multiple transcripts that had been annotated, we counted the numbers of new TE insertions in each region for each alternative transcript and then took the average over transcripts.

## Supporting Information

Figure S1 The relationship between tissue expression profiles. The square of the Euclidean distance was calculated between the log of the relative abundance values between tissue expression profiles across genes, and a phylogenetic tree then constructed using neighbour joining.
(0.36 MB TIF)

Figure S2 The relationship between the square of the Euclidean distance and time, in simulations, when relative abundance values are calculated. Two examples are shown. In both there are 1000 genes which are given an initial random expression profile across tissues. The random expression profile is generated such that the log expression value is normally distributed with a mean of zero and standard deviation of one. In each generation a normal random deviate with a mean of zero and standard deviation of 0.1, was added to the log expression values. The simulation was run until the expression divergence was twice as high as the average expression divergence seen in our data. The upper line is for 10 tissues, the bottom line for 2 tissues.
(0.12 MB TIF)

Figure S3 A geometrical argument showing that Z, the effect of TEs on ED, is underestimated when the regression is performed on the Box-Cox transformed data. The red lines indicate the real the relationship between the square of the Euclidean distance and the number of TE insertions. The blue line represents the linear regression performed on the Box-Cox transformed data. See text for further explanation.
(0.14 MB TIF)

Text S1 File gives evidence that brain tissues tend to evolve in concert between mouse and rat, and proves two results pertaining to the measurement of expression divergence
(0.05 MB DOC)
