# Supplementary figures and images for: The Effect of Transposable Element Insertions on Gene Expression Evolution in Rodents
Source: PLoS One. 2009 Feb 2;4(2):e4321. doi: 10.1371/journal.pone.0004321 (PMC2629548; doi:10.1371/journal.pone.0004321)

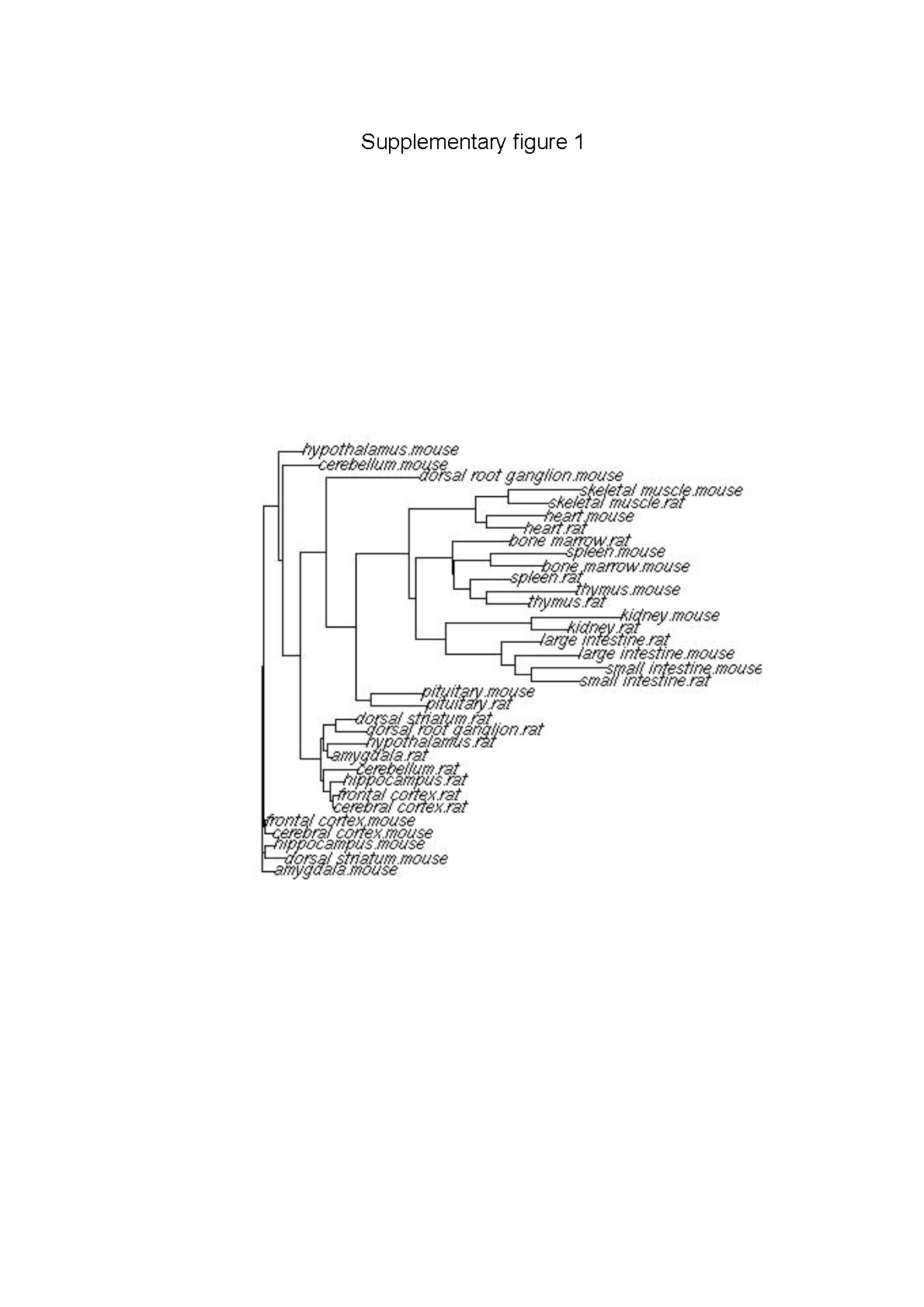

Supplement: Figure S1 — The relationship between tissue expression profiles. The square of the Euclidean distance was calculated between the log of the relative abundance values between tissue expression profiles across genes, and a phylogenetic tree then constructed using neighbour joining. (0.36 MB TIF) [file pone.0004321.s001.tif]

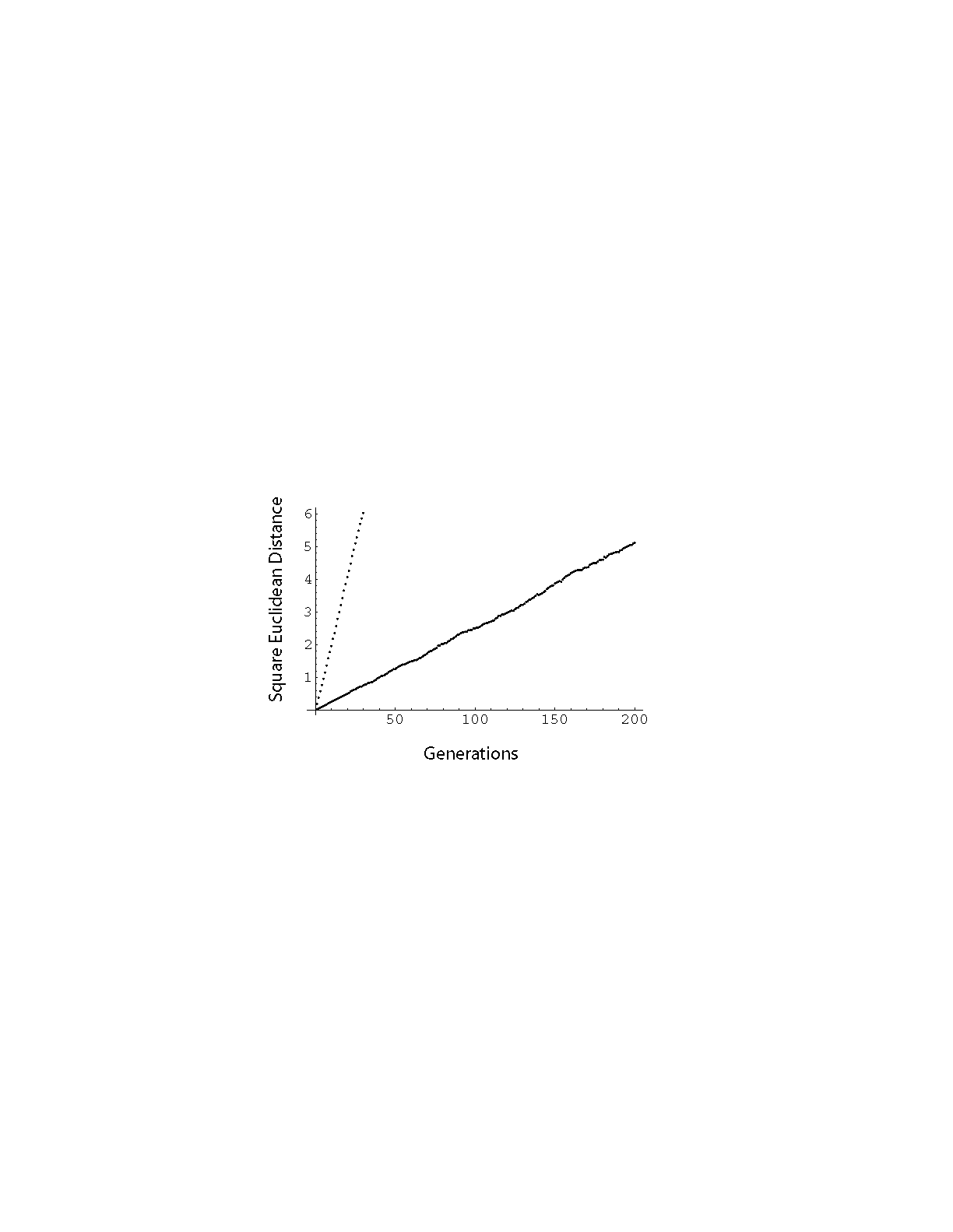

Supplement: Figure S2 — The relationship between the square of the Euclidean distance and time, in simulations, when relative abundance values are calculated. Two examples are shown. In both there are 1000 genes which are given an initial random expression profile across tissues. The random expression profile is generated such that the log expression value is normally distributed with a mean of zero and standard deviation of one. In each generation a normal random deviate with a mean of zero and standard deviation of 0.1, was added to the log expression values. The simulation was run until the expression divergence was twice as high as the average expression divergence seen in our data. The upper line is for 10 tissues, the bottom line for 2 tissues. (0.12 MB TIF) [file pone.0004321.s002.tif]

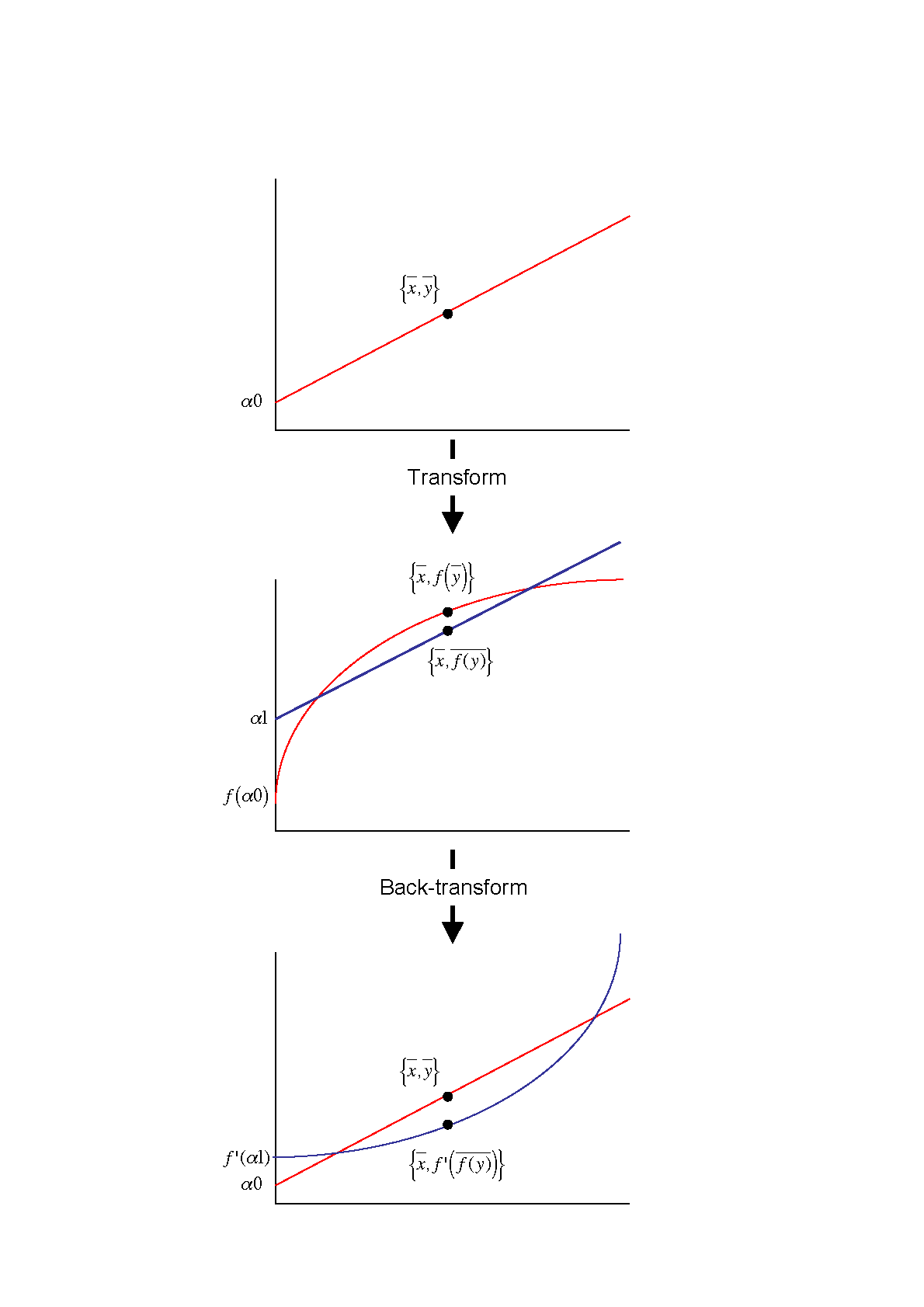

Supplement: Figure S3 — A geometrical argument showing that Z, the effect of TEs on ED, is underestimated when the regression is performed on the Box-Cox transformed data. The red lines indicate the real the relationship between the square of the Euclidean distance and the number of TE insertions. The blue line represents the linear regression performed on the Box-Cox transformed data. See text for further explanation. (0.14 MB TIF) [file pone.0004321.s003.tif]
