# Supplementary material for: The Effect of Transposable Element Insertions on Gene Expression Evolution in Rodents
Source: PLoS One. 2009 Feb 2;4(2):e4321. doi: 10.1371/journal.pone.0004321 (PMC2629548; doi:10.1371/journal.pone.0004321)
Supplement: Text S1 — File gives evidence that brain tissues tend to evolve in concert between mouse and rat, and proves two results pertaining to the measurement of expression divergence (0.05 MB DOC) [file pone.0004321.s004.doc]

**The effect of Transposable Element Insertions on Gene Expression Evolution in Rodents**

Vini Pereira, David Enard and Adam Eyre-Walker

**Supplementary information**

Here we provide evidence for two results which allow us to estimate the average effect of TE insertion upon expression divergence. First, we show that the square of the Euclidean distance is expected to increase linearly with time under a simple random walk model. Second, we show we are likely to underestimate the effect of TEs on ED when we perform the regression on the Box-Cox transformed data, and then back transform the mean and intercept to the square of the Euclidean distance.

Khaitovich et al. [1] have previously shown the squared difference in the log expression values for a single gene in single tissue is expected to increase linearly with time under a simple random walk model in which changes in log expression are normally distributed (this is equivalent to fold changes in expression level being log-normally distributed). It is therefore evident that the square in the Euclidean distance between the log-expression level of a single gene across multiple tissues will also scale linearly with time under the same random walk model; this is because the square of the Euclidean distance is a sum of the squared differences.

However, we have used relative abundance (RA) values to control for probe effects; i.e. we have divided the expression level of a gene in a tissue by the sum of the expression of that gene across all tissues. To investigate whether the Euclidean distance between log RA values also scales linearly with time we performed several simulations in which gene expression profiles were subject to a random walk, with RA values being calculated prior to the calculation of the Euclidean distance. To be precise we took the log expression values and added to these a random normal deviate with a mean of zero and standard deviation of σ. We then took the exponent of these values, calculated the RA values and took the logarithm of the result before calculating the square of the Euclidean distance. We performed simulations in which the initial expression profile was randomly generated or uniform; we considered cases in which we had 2 and 10 tissues. We also took the expression profiles from the 3072 genes from the rat or mouse as the initial state. We allowed these to evolve using different values of σ. In all cases the relationship between the square of the Euclidean distance between log RA values was linear with time. Some examples are shown in Figure S2.

We have performed our regression analysis on the Box-Cox transformed Euclidean distance, rather than the Euclidean distance or square of the Euclidean distance, because neither the Euclidean distance or its square is normally distributed. In fact both have very skewed distributions which means that some points have large influences on the regression model. As a consequence we have calculated *Z* by taking the mean and intercept from our regression model and back-transforming these to the square of the Euclidean distance before calculating *Z*. A simple geometrical argument suggests that doing this will lead to an underestimate of *Z* (Figure S3)*.* Consider a series of points {*x_1_, y_1_*}…{*x_k_, y_k_*} which lie on a straight line, which has intercept of α_0_. Imagine that we now apply a transformation, $f(y)$, to the *y*-variable such that the line becomes concave; for example we might log-transform the variable or take the square-root. Note that . If we perform linear regression we also note that α_1_ > *f*(*α_0_*) where *α_1_* is the intercept from the linear regression. Hence if we back transform the mean and intercept using the function *f’(x)* we know that and *f’*(*α_1_*) > *α_0_*, hence .

We confirmed this reasoning by simulating data that lay on a straight line; we then took the square-root and applied the Box-Cox transformation before performing the linear regression. We back-transformed the intercept from the regression model and the mean of the Box-Cox transformed data, before calculating Z. We performed this analysis for various slopes and intercepts. In all cases Z was underestimated.

**Literature cited**

1. Khaitovich P, Weiss G, Lachmann M, Hellmann I, Enard W, et al. (2004) A neutral model of transcriptome evolution. PLoS Biol 2: E132.

**Figure S1.** The relationship between tissue expression profiles. The square of the Euclidean distance was calculated between the log of the relative abundance values between tissue expression profiles across genes, and a phylogenetic tree then constructed using neighbour joining.

**Figure S2.** The relationship between the square of the Euclidean distance and time, in simulations, when relative abundance values are calculated. Two examples are shown. In both there are 1000 genes which are given an initial random expression profile across tissues. The random expression profile is generated such that the log expression value is normally distributed with a mean of zero and standard deviation of one. In each generation a normal random deviate with a mean of zero and standard deviation of 0.1, was added to the log expression values. The simulation was run until the expression divergence was twice as high as the average expression divergence seen in our data. The upper line is for 10 tissues, the bottom line for 2 tissues.

**Figure S3.** A geometrical argument showing that *Z*, the effect of TEs on ED, is underestimated when the regression is performed on the Box-Cox transformed data. The red lines indicate the real the relationship between the square of the Euclidean distance and the number of TE insertions. The blue line represents the linear regression performed on the Box-Cox transformed data. See text for further explanation.
